# Vitamin D status in children with COVID-19: does it affect the development of long COVID and its symptoms?

**DOI:** 10.3389/fped.2025.1507169

**Published:** 2025-02-14

**Authors:** Vita Perestiuk, Tetyana Kosovska, Olha Dyvoniak, Liubov Volianska, Oksana Boyarchuk

**Affiliations:** ^1^ Department of Children’s Diseases and Pediatric Surgery, I.Horbachevsky Ternopil National Medical University, Ternopil, Ukraine; ^2^ Department of Pediatric Infectious Diseases, Ternopil City Hospital N2, Ternopil, Ukraine

**Keywords:** COVID-19, SARS-CoV-2 infection, long COVID, vitamin D, 25(OH)D, children vitamin D

## Abstract

**Introduction:**

Long COVID is characterized by diverse symptoms persisting after severe acute respiratory syndrome coronavirus 2 (SARS-CoV-2). Given the immunomodulatory and neuroprotective properties of vitamin D, understanding its role in long COVID symptoms is of growing interest. This study aimed to determine vitamin D status in children with COVID-19 and assess its impact on the clinical course of disease and long COVID development.

**Methods:**

A prospective cohort study included hospitalized children with confirmed COVID-19, aged 1 month to 18 years, diagnosed between September 2022 and March 2024. Serum 25-hydroxyvitamin D (25(OH)D) concentrations were measured upon hospital admission, and follow-up was done to identify long COVID symptoms.

**Results:**

In total, 162 hospitalized patients with COVID-19 were examined. Vitamin D deficiency was determined in 8.0%, insufficiency in 25.3%, and optimal levels in 66.7% of children with COVID-19. Vitamin D deficiency/insufficiency was observed in 73% of children over 6 years and 21.6% of children under 6 years of age. Comorbid conditions were 1.4 times more frequent in children with vitamin D insufficiency, with undernutrition and obesity playing the most significant roles (*p* = 0.0023, *p* = 0.0245, respectively). Serum 25(OH)D concentration depends on COVID-19 severity (*p* = 0.0405) and children with vitamin D deficiency/insufficiency had a longer hospital stay (4 vs. 3 days, *p* = 0.0197). The vitamin D status affected the median levels of neutrophils, lymphocytes, their ratio, prothrombin time, fibrinogen levels, and the frequency of increased immunoglobulins M and E levels. Among 134 children who agreed to follow up, 56 (41.8%) experienced long COVID symptoms, while 78 (58.2%) recovered fully. Long COVID was frequently observed in children with vitamin D deficiency/insufficiency (*p* = 0.0331). The odds of developing long COVID were 2.2 times higher (*p* = 0.0346) in children with vitamin D deficiency/insufficiency compared to those with optimal levels. Children with vitamin D deficiency/insufficiency more often exhibited neurological (80% vs. 41.9%, *p* = 0.0040) and musculoskeletal symptoms (16% vs. 0%, *p* = 0.0208).

**Conclusion:**

The 25(OH)D concentrations in children with COVID-19 depended on their age. Comorbid conditions affect the vitamin D status in children with COVID-19. Vitamin D influenced the COVID-19 severity and duration of hospitalization. There was an increased risk of developing long COVID in children with vitamin D deficiency/insufficiency, and its impact on the development of neurological symptoms associated with long COVID was established.

## Introduction

COVID-19, caused by severe acute respiratory syndrome coronavirus 2 (SARS-CoV-2), primarily affects the respiratory system, leading to conditions like interstitial pneumonia and acute respiratory distress syndrome (1). Although COVID-19 can cause severe complications in adults, especially those with comorbidities, most children experience mild or asymptomatic cases, with very few requiring hospitalization and a low mortality rate globally (2, 3).

As the incidence of SARS-CoV-2 infection increases, concerns are rising regarding persistent symptoms following acute infection, widely known as “long COVID” (4). Long COVID (sometimes referred to as “post-acute sequelae of COVID-19” or “post-COVID-19 syndrome”) is a multisystem condition characterized by a variety of symptoms, including cardiorespiratory, neurological, psychosomatic, sensory, cognitive, and psychological symptoms that arise after infection with coronavirus type 2 (4, 5). As of September 1, 2024, the World Health Organization (WHO) reports that more than 776 million cases of COVID-19 have been documented worldwide, and 10%–30% of non-hospitalized individuals and 50%–70% of hospitalized patients may experience long COVID (6, 7).

Long COVID raises growing health concerns as its persistence can affect multiple organ systems with potentially negative impacts on quality of life (8, 9). Only a few prospective studies collect systematic data in larger cohorts of children with multidisciplinary clinical assessment (4, 5, 10, 11).

Post-COVID syndrome in adults is more often associated with prolonged tissue damage following persistent inflammation caused by the virus, immune dysregulation, autoimmune processes, endothelial damage, and microthrombosis (12, 13). In children and adolescents, similar pathogenic mechanisms are plausible, but the significance of vitamin and micronutrient deficiencies is also being discussed (14). In developing long COVID symptoms in children, age, certain comorbid conditions, and hospitalization in intensive care units were considered important factors (14–16).

Despite the importance of control measures during the COVID-19 pandemic (17–19), prolonged stay at home negatively impacted the health and development of children (20). Children who remain at home for extended periods are more prone to physical inactivity, unhealthy diets, and limited sunlight exposure, which may put them at greater risk of vitamin D deficiency and insufficiency (20–22).

Vitamin D plays a key role in maintaining calcium homeostasis and bone health, as well as having immunoregulatory effects as a potent regulator of innate and adaptive immune responses, influencing the expression of antimicrobial peptides and the inflammatory cascade (23–25). It has been shown to influence gene expression, modulating immune response, inflammation, oxidative stress, and the gut microbiota (26). Optimal serum vitamin D levels are fundamental for promoting health in both pediatric and adult populations (27).

Several studies have shown a connection between symptoms, severity, mortality, and outcomes of COVID-19 and vitamin D concentration in patients, regardless of age (28, 29). However, the majority of studies are related to the adult population. Szerszeń et al. (30) noted a correlation between patient mortality, the need for oxygen therapy, and vitamin D levels in older patients. Some publications have shown the impact of vitamin D status on the development of long COVID, including multisystem inflammatory syndrome (31). However, the studies on the significance of vitamin D in the development and course of COVID-19 in children, and its role in the emergence of long COVID in the pediatric population remain limited and their results are contradictory. The aim of our study was to determine vitamin D status in children with COVID-19 and assess its impact on the clinical course of disease and long COVID development.

## Materials and methods

### Study design

A prospective cohort study was conducted from September 2022 to March 2024 in the pediatric infectious diseases department of a tertiary-level hospital in Ternopil, Ukraine.

### Participants

The study included hospitalized patients aged 1 month to 18 years diagnosed with COVID-19. All cases of SARS-CoV-2 infection were confirmed using polymerase chain reaction (PCR), rapid tests, or serological methods (detection of class M antibodies).

The inclusion criteria for the study were age up to 18 years, confirmed cases of SARS-CoV-2 infection, informed consent from parents or patients, and the ability to determine the concentration of 25(OH)D in serum during hospitalization. Exclusion criteria included parental refusal for examination and unconfirmed cases of COVID-19.

### Data collection and laboratory assessments

A thorough collection of baseline and clinical data was conducted upon patient admission. The baseline characteristics included age and sex, while the clinical signs encompassed comorbidities, the severity of COVID-19, and the duration of hospitalization. The severity of COVID-19 was determined according to the WHO definition (32).

A comprehensive laboratory examination at admission included a complete blood count, determination of biochemical blood analysis indicators, coagulogram, and serum immunoglobulins (Ig) A, M, G, and E. Serum immunoglobulins were determined using the Monobind enzyme-linked immunosorbent assay (ELISA) kit, AccuBind ELISA Kits, USA. The evaluation of immunoglobulin levels was conducted according to age-specific norms.

To assess vitamin D status, its quantitative measurement was conducted in serum. For this purpose, the concentration of 25-hydroxyvitamin D (25(OH)D) in serum was determined using the ELISA, FCCu Bind ELISA Microwells, USA.

According to the recommendations of the European Vitamin D Association (EVIDAS), a concentration of 25(OH)D between 30 and 100 ng/ml (75–250 nmol/L) was considered optimal; 20–30 ng/ml (50–75 nmol/L) was classified as vitamin D insufficiency; and concentrations below 20 ng/ml (<50 nmol/L) indicated vitamin D deficiency (33).

### Long COVID definition and monitoring

After discharge from the hospital, patients were monitored for the presence of long-term symptoms of COVID-19. For this purpose, we conducted surveys at intervals of 1–3, 3–6, 6–9, and 9–12 months after the acute phase of infection, using the questionnaire developed by the International Severe Acute Respiratory and Emerging Infection Consortium (ISARIC)/IP4C Global Pediatric COVID-19 Follow-Up Form. Patients or their parents, in cases where the children were under 8 years old, answered the questions. The presence of “long COVID” was determined according to WHO criteria, defined as the continuation or development of new symptoms at least 3 months after the initial SARS-CoV-2 infection, with a duration of at least 2 months with no other explanations (6).

**Figure 1:**
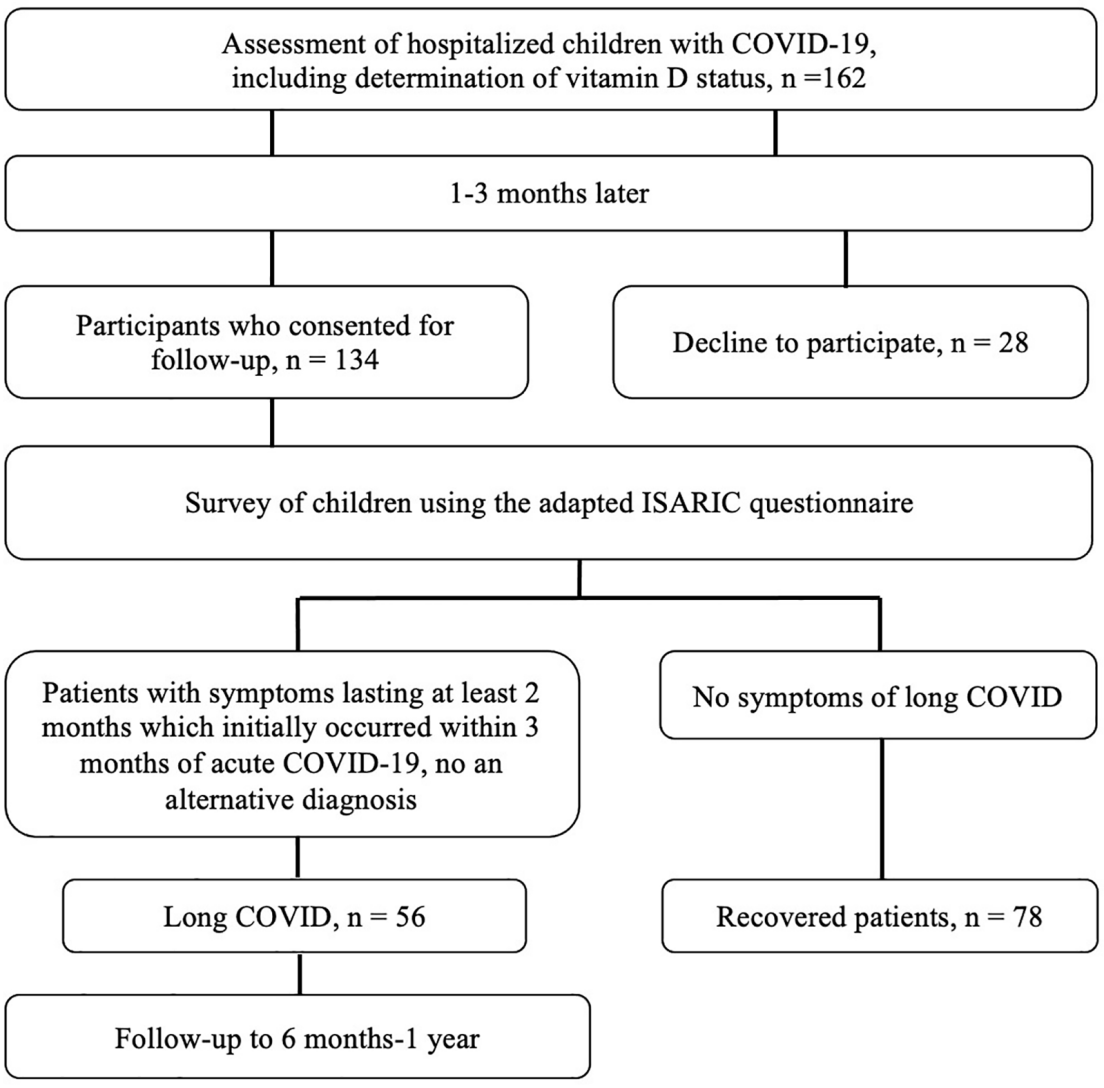
Patients flow diagram.

Patients who did not have symptoms during the follow-up period after the onset of acute COVID-19 symptoms for at least 8 weeks were defined as fully recovered. The selection of patients for the study is shown in Figure 1.

### Ethical considerations

Throughout our study, we adhered to all recommendations of the 1975 Declaration of Helsinki (as revised in 2000). The study was approved by the I. Horbachevsky Ternopil National Medical University Ethics Committee (Minutes № 70 from August 1, 2022). Upon admission, all parents or children who reached the age of 16 signed an informed, voluntary consent for the study, as well as for the use of diagnostic and treatment results in scientific works.

### Statistical analysis

Statistical analysis of the results was performed using the STATISTICA 12 software. All data were described as the mean ± standard deviation (SD) for normally distributed data or median and interquartile range (IQR) for skewed distributions and categorical variables expressed as frequency (percentage). Differences in variables with a normal distribution between two independent samples were compared using the Student's t-test, while the results with a non-normal distribution were analyzed using the Mann–Whitney *U*-test, and categorical variables were compared using the Chi-square test. A *p*-value of less than 0.05 was defined as statistically significant and highlighted in the tables in bold font.

Odds ratio (OR) and 95% confidence intervals (CI) were determined to explore the influence of vitamin D deficiency on the development of long COVID. For this purpose, we used only statistically significant features.

## Results

### Characteristics of children with COVID-19

In total, 162 hospitalized patients with COVID-19 were examined. Clinical and laboratory characteristics of children with COVID-19 are presented in Table 1. The average age of hospitalized patients with COVID-19 was 3.62 ± 4.55 years, ranging from 1 month to 18 years. Boys predominated over girls in the overall cohort of patients (56.8%).

**Table 1:** Clinical characteristics of the patients with COVID-19 and their dependence on vitamin D status.

Characteristics	Total, *n* = 162	Vitamin D deficiency/insufficiency, *n* = 54	Optimal vitamin D level, *n* = 108	*P*
	Median (interquartile range, IQR) or *n* (%)			
Age of children, years	1.3 (0.7;5.33)	5.91 (2.08;11.0)	0.82 (0.53;1.5)	**<0** **.** **0001**
Gender				
Female	70 (43.2)	28 (51.9)	42 (38.9)	0.1164
Male	92 (56.8)	26 (48.1)	66 (61.1)	0.1164
Comorbid conditions:	75 (46.3)	31 (57.4)	44 (40.7)	**0**.**0449**
Allergic diseases	36 (22.2)	9 (16.7)	27 (25.0)	0.2291
Nutritional disorders	42 (25.9)	22 (40.7)	20 (18.5)	**0**.**0023**
Overweight	15 (9.3)	8 (14.8)	7 (6.5)	0.0845
Obesity	13 (8.0)	8 (14.8)	5 (4.6)	**0**.**0245**
Undernutrition	14 (8.6)	6 (11.1)	8 (7.4)	0.4290
Cardiovascular pathologies	10 (6.2)	2 (3.7)	8 (7.4)	0.3558
Nervous system diseases	16 (9.9)	5 (9.3)	11 (10.2)	0.8523
Digestive system diseases	7 (4.3)	4 (7.4)	3 (2.8)	0.1719
Urinary system diseases	5 (3.1)	2 (3.7)	3 (2.8)	0.7480
COVID-19 severity				
Mild	146 (90.1)	47 (87.0)	99 (91,7)	0.4056
Moderate	7 (4.3)	4 (7.4)	3 (2.8)	0.2232
Severe/critical	9 (5.6)	3 (5.6)	6 (5.6)	1.0000
Duration of hospitalization, days	4.0 (3.0; 5.0)	4.0 (3.0; 6.0)	3.0 (3.0; 5.0)	**0**.**0197**
Leukocytes, 10^9^/L	5.94 (4.44; 8.8)	5.93 (4.37; 8.44)	5.94 (4.53; 8.89)	0.7979
Leukocytosis	16/161 (9.9)	7/54 (13.0)	9/107 (8.4)	0.4074
Neutrophils, 10^9^/L	2.37 (1.33; 3.69)	3.1 (2.13; 4.06)	1.88 (1.06; 3.25)	**0**.**0021**
Neutrophilia	13/157 (8.3)	6/51 (11.8)	7/106 (6.6)	0.2718
Lymphocytes, 10^9^/L	2.12 (1.21; 3.99)	1.65 (0.97; 2.56)	2.38 (1.42; 4.41)	**0**.**0044**
Lymphopenia	84/157 (53.5)	25/51 (49.0)	59/106 (55.7)	0.4346
Neutrophil-to-lymphocyte ratio	1.16 (0.44; 2.64)	2.19 (1.09; 3.5)	0.83 (0.34; 2.08)	**<0**.**0001**
More than 4	20/157 (12.7)	10/51 (19.6)	10/106 (9.4)	0.0734
Thrombocytes, 10^9^/L	244.0 (204.0; 310.0)	218.5 (187.0; 263.0)	264.5 (208.0;323.0)	**0**.**0071**
Thrombocytopenia, %	11/161 (6.8)	3/54 (5.6)	8/107 (7.5)	0.7522
CRP, mg/L	5.48 (1.41; 15.9)	5.86 (3.4; 20.8)	5,25 (1.4; 14.5)	0.1259
Elevated CRP, %	79/145 (54.5)	30/49 (61.2)	49/96 (51.0)	0.2914
Prothrombin time (PT), sec	14.7 (13.4; 15.9)	15.5 (13.8; 16.4)	14.5 (13.3; 15.6)	**0**.**0058**
Prolonged PT (more than 15 s)	3/147 (42.9)	31/49 (63.3)	32/98 (32.7)	**0**.**0007**
Activated partial thromboplastin time (aPTT), sec	38.3 (34.0; 44.0)	37.1 (32.2; 41.7)	39.6 (34.8; 45.3)	0.1074
Prolonged aPTT (more than 35 s)	98/147 (66.7)	30/49 (61.2)	68/98 (69.4)	0.3563
Fibrinogen, g/L	2.17 (1.67; 2.98)	2.66 (1.97; 3.42)	2.0 (1.52; 2.86)	**0**.**0037**
More than 4 g/L	8/139 (5.8)	4/45 (8.9)	4/94 (4.3)	0.2734
D-dimer, ng/ml	270.0 (100; 750)	239.0 (90; 390)	360.5 (150; 840)	0.1269
More than 250 ng/ml	30/59 (50.9)	10/25 (40.0)	20/34 (58.8)	0.1923
Alanine aminotransferase (ALT), U/L	21.0 (15.0; 30.0)	15.0 (12.0; 19.0)	24.0 (18.0; 33.0)	**<0**.**0001**
Elevated ALT level, %	11/155 (7.1)	3/51 (5.9)	8/104 (7.7)	1.000
Ferritin	49.1 (27.8; 88.9)	47.29 (25.2; 86.6)	49.15 (33.81; 118,96)	0.4088
Hyperferritinemia, %	2/48 (4.2)	0/20 (0)	2/28 (7.1)	0.5035
Immunoglobulin A				
Optimal level	32/56 (57.1)	13/29 (44.8)	19/27 (70.4)	0.0644
Increase level	24/56 (42.9)	16/29 (55.2)	8/27 (29.6)	0.0644
Immunoglobulin M				
Optimal level	17/56 (30.4)	6/29 (20.7)	11/27 (40.7)	0.1475
Decrease level	2/56 (3.6)	0	2/27 (7.4)	0.2279
Increase level	37/56 (66.1)	23/29 (79.3)	14/27 (51.9)	**0**.**0476**
Immunoglobulin G				
Optimal level	41/56 (73.2)	19/29 (65.6)	22/27 (81.5)	0.2329
Decrease level	7/56 (12.5)	5/29 (17.2)	2/27 (7.4)	0.4239
Increase level	8/56 (14.3)	5/29 (17.2)	3/27 (11.1)	0.7066
Immunoglobulin E				
Optimal level	38/54 (70.4)	16/29 (55.2)	22/25 (88.0)	**0**.**0154**
Increase level	16/54 (29.6)	13/29 (44.8)	3/25 (12.0)	**0**.**0154**

Statistically significant values are highlighted in bold.

Comorbidities were present in 75 (46.3%) patients, with 31 children (19.1%) having two or more conditions. Nutritional disorders were the most common comorbidities (25.9%), followed by allergic conditions (22.2%). Disorders of the nervous, cardiovascular, gastrointestinal systems, and kidneys were less frequently observed (Table 1).

Children with mild COVID-19 predominated in the cohort (90.1%). Moderate cases were observed in 4.3%, severe cases in 4.3%, and critical cases in 1.2% of patients. Eight patients (4.9%) developed COVID-19-related pneumonia. Seven patients (4.3%) required treatment in the intensive care unit, and four children (2.5%) required oxygen therapy during their treatment. Oxygen therapy was administered using a nasal cannula or a face mask, with the duration of oxygen supply depending on the effectiveness of the therapy and the severity of respiratory disorders. The average duration of oxygen therapy was 3 days. Mechanical ventilation was provided briefly for two children who developed acute respiratory failure. No deaths were reported among the children in this cohort. The average length of hospitalization was 4.6 ± 3.0 days, ranging from 1 to 20 days.

Leukocytosis was observed in 9.9% of children, lymphopenia in 53.5%, and neutrophilia in 8.3% of patients. A neutrophil-to-lymphocyte ratio greater than 4 was noted in 12.7% of patients. In most children with COVID-19 (85.7%), platelet counts were within the normal range. An elevated C-reactive protein (CRP) level was found in 54.5% of patients.

A reduced fibrinogen level was identified in 42.5% of patients, while an elevated fibrinogen level was noted in 5.8%. D-dimer levels were elevated in 50.9% of patients upon admission. Ferritin levels ranged from 4.03 to 440 ng/ml, with elevated levels observed in only two cases (4.2%).

An increase in IgA was observed in 42.9% of children, IgM in 66.1%, IgG in 14.3%, and IgE in 29.6% of children. Additionally, decreased levels of IgM were detected in 3.6% of children and IgG in 12.5% of children.

### Vitamin D status in patients with the acute phase of SARS-CoV-2 infection

**Figure 2:**
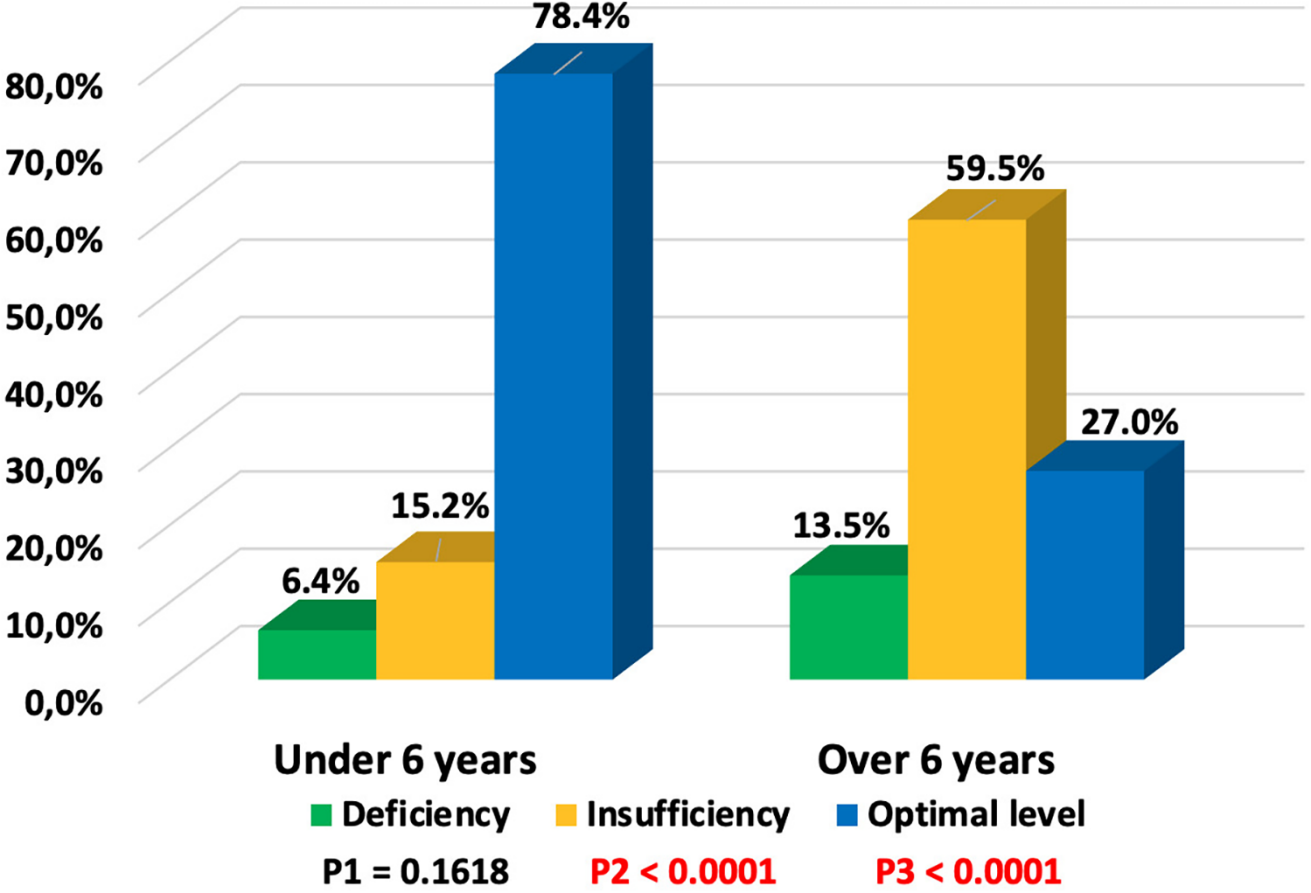
Dependence of vitamin D status on age in children with COVID-19 (P1—value between groups under 6 years and over 6 years with vitamin D deficiency, P2—value between groups under 6 years and over 6 years with vitamin D insufficiency, P3—value between groups under 6 years and over 6 years with optimal vitamin D levels).

**Figure 3:**
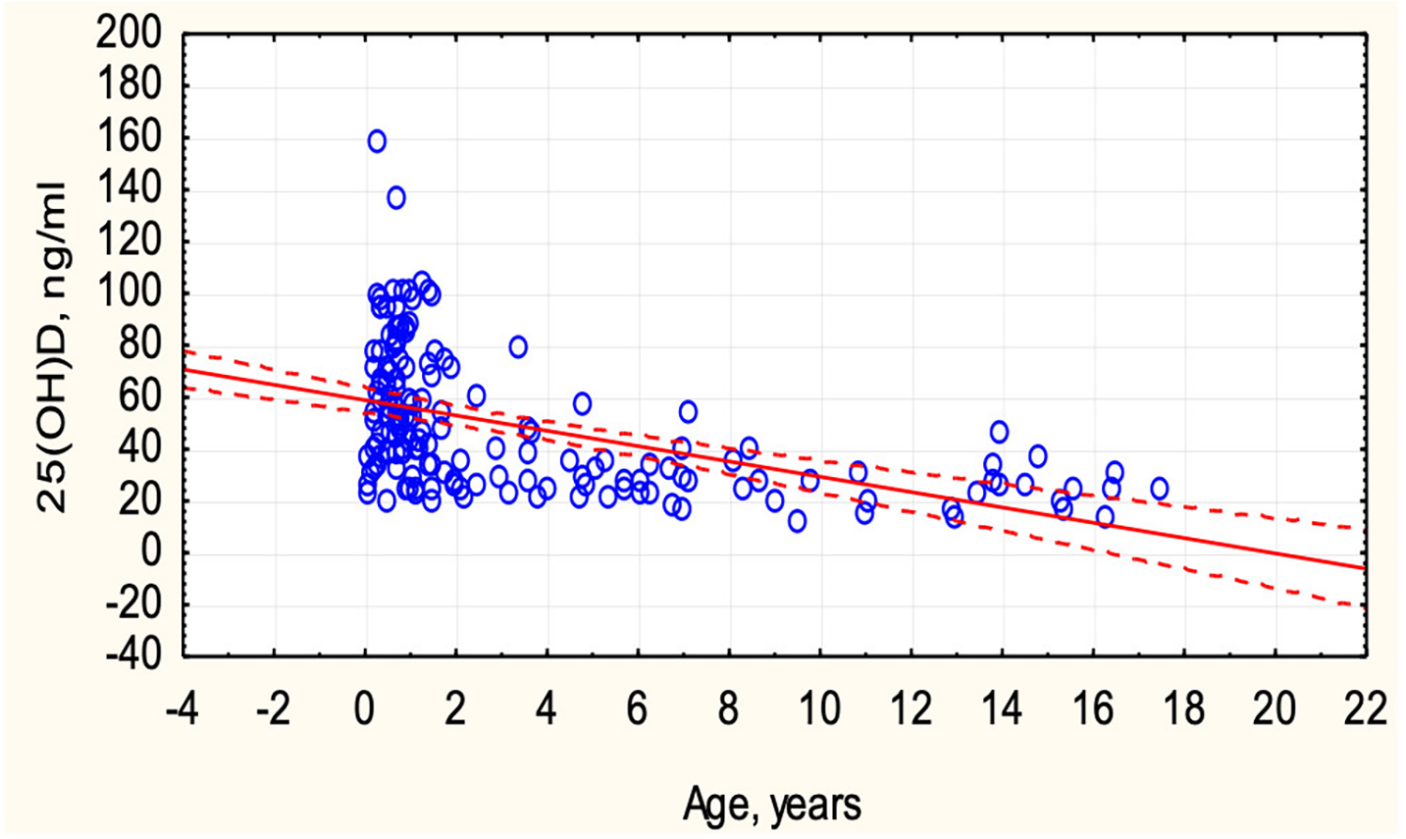
Correlation between serum 25(OH)D concentration and children's age.

Vitamin D deficiency was identified in 13 (8.0%), insufficiency in 41 (25.3%), and optimal levels in 108 (66.7%) children with COVID-19. Vitamin D status depended on the age of the children (Figure 2). In children under 6 years old, optimal vitamin D levels were more frequently observed (78.4% vs. 21.6%, *p* < 0.0001), while in children over 6 years, only 27% had optimal levels, and 73% exhibited deficiency and insufficiency. An inverse correlation was observed between the concentration of 25(OH)D and the age of the children (r = −0.4989, *p* < 0.05) (Figure 3).

### Comparison of clinical characteristics of COVID-19 based on vitamin D status

In the first group, with 25(OH)D concentrations below 30.0 ng/ml, there were 54 (33.3%) children, while the remaining 108 (66.7%) were classified in the second group with optimal vitamin D levels (30.0–100.0 ng/ml). The comparison of clinical characteristics of COVID-19 patients based on vitamin D status is presented in Table 1.

Overall, comorbid conditions were 1.4 times more prevalent in children with vitamin D insufficiency, and this difference was statistically significant (*p* = 0.0449). Specifically, nutritional disorders, particularly obesity, were significantly more common in children with low vitamin D levels (*p* = 0.0023 and *p* = 0.0245, respectively).

**Figure 4:**
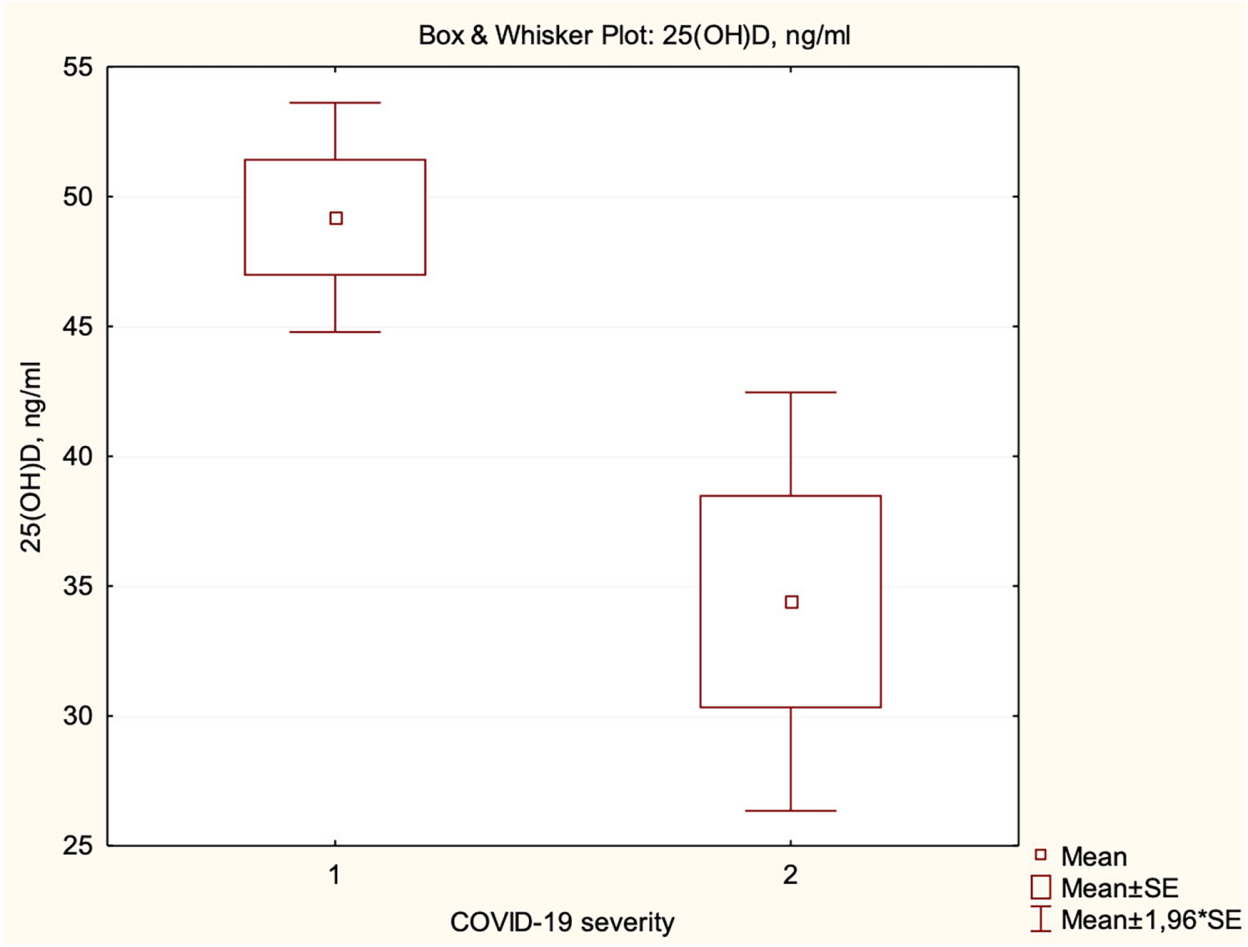
Dependence of serum 25(OH)D concentration on the severity of COVID-19 (1—mild, 2—moderate and severe/critical).

We did not find a correlation between the severity of COVID-19 and vitamin D status. However, the mean level of serum 25(OH)D concentration in children with mild course was significantly higher than in children with moderate and severe/critical course (49.19 ng/ml vs. 34.40 ng/ml, *p* = 0.0405) (Figure 4). Children with vitamin D deficiency and insufficiency had longer hospital stays (4 vs. 3 days, *p* = 0.0197).

The leukocyte level, as well as the percentage of children with leukocytosis, did not depend on vitamin D status in COVID-19 patients. The median neutrophil level was higher in the group of patients with low vitamin D levels (*p* = 0.0021), while the median lymphocyte level was higher in patients with optimal vitamin D levels (*p* = 0.0044). When examining the ratio of neutrophils to lymphocytes, it was found that the ratio was lower in the group with normal vitamin D levels, *p* < 0.0001. However, the percentage of children with neutrophilia, lymphopenia, and an elevated neutrophil-to-lymphocyte ratio did not differ between the two groups, which may indicate a greater age dependence, as these indicators change with age, and considering that most children under 6 years had optimal vitamin D levels, while those over 6 predominantly had vitamin D insufficiency. However, the number of children with a neutrophil-to-lymphocyte ratio greater than 4 was twice as high among those with vitamin D deficiency/insufficiency, *p* = 0.0734. In patients with optimal vitamin D levels, the median platelets count was significantly higher (*p* = 0.0071), but there was no significant difference in the frequency of thrombocytopenia (*p* = 0.6587).

The CRP level, although somewhat higher in the group of patients with low vitamin D levels, did not differ significantly (Table 1).

Only 11 out of 146 (7.5%) hospitalized children with COVID-19 had all coagulation parameters assessed in this study within the normal range. The medians of PT and fibrinogen were higher in the cohort of patients with low vitamin D levels (*p* = 0.0058 and *p* = 0.0037, respectively). The proportion of children with prolonged PT and elevated fibrinogen levels was twice as high in patients with vitamin D deficiency/insufficiency and COVID-19, although the difference was statistically significant only for PT. The median values of aPTT and D-dimer were not dependent on vitamin D status.

The median ALT level was higher in children with optimal vitamin D levels; however, the proportion of children with elevated ALT and ferritin levels did not differ between the two groups of children with COVID-19 (Table 1).

When comparing immunoglobulin levels in children with deficient or insufficient vitamin D levels to those with optimal vitamin D levels, it was found that children with vitamin D deficiency/insufficiency more frequently exhibited elevated levels of IgA (55.2% vs. 29.6%), IgM (79.3% vs. 51.9%), IgG (17.2% vs. 11.1%), and IgE (44.8% vs. 12.0%). The differences were statistically significant for IgM (*p* = 0.0476) and IgE (*p* = 0.0154), with a trend toward increased IgA levels (*p* = 0.0644).

### Clinical characteristics of children with long COVID

Of the 162 patients included in the study, 134 consented to further observation. Among them, symptoms of long COVID were identified in 56 (41.8%) children, while 78 (58.2%) recovered fully. The observation period ranged from 6 months to 1 year, with an average of 10.4 months. Among the symptoms in children with long COVID, general manifestations predominated, observed in 37 (66.1%) children. These included fatigue, general weakness, decreased appetite, reduced physical activity, and difficulties starting tasks. Neurological symptoms included insomnia or excessive sleepiness, headache, increased irritability, emotional lability, decreased memory and attention, and inability to concentrate on tasks. This group of symptoms was present in 33 (58.9%) children. Gastroenterological symptoms, such as hepatopathy, abdominal pain, nausea, and constipation, were observed in 9 (16.1%) patients. Cardiological manifestations, including tachycardia or conduction disturbances, were less common, occurring in 2 (3.6%) children, while musculoskeletal symptoms (myalgias and arthralgias) were noted in 4 patients (7.1%). Additionally, 20 (35.7%) children experienced frequent acute respiratory infections, which occurred significantly more often than before the SARS-CoV-2 infection.

### Long COVID symptoms depend on vitamin D status

**Figure 5:**
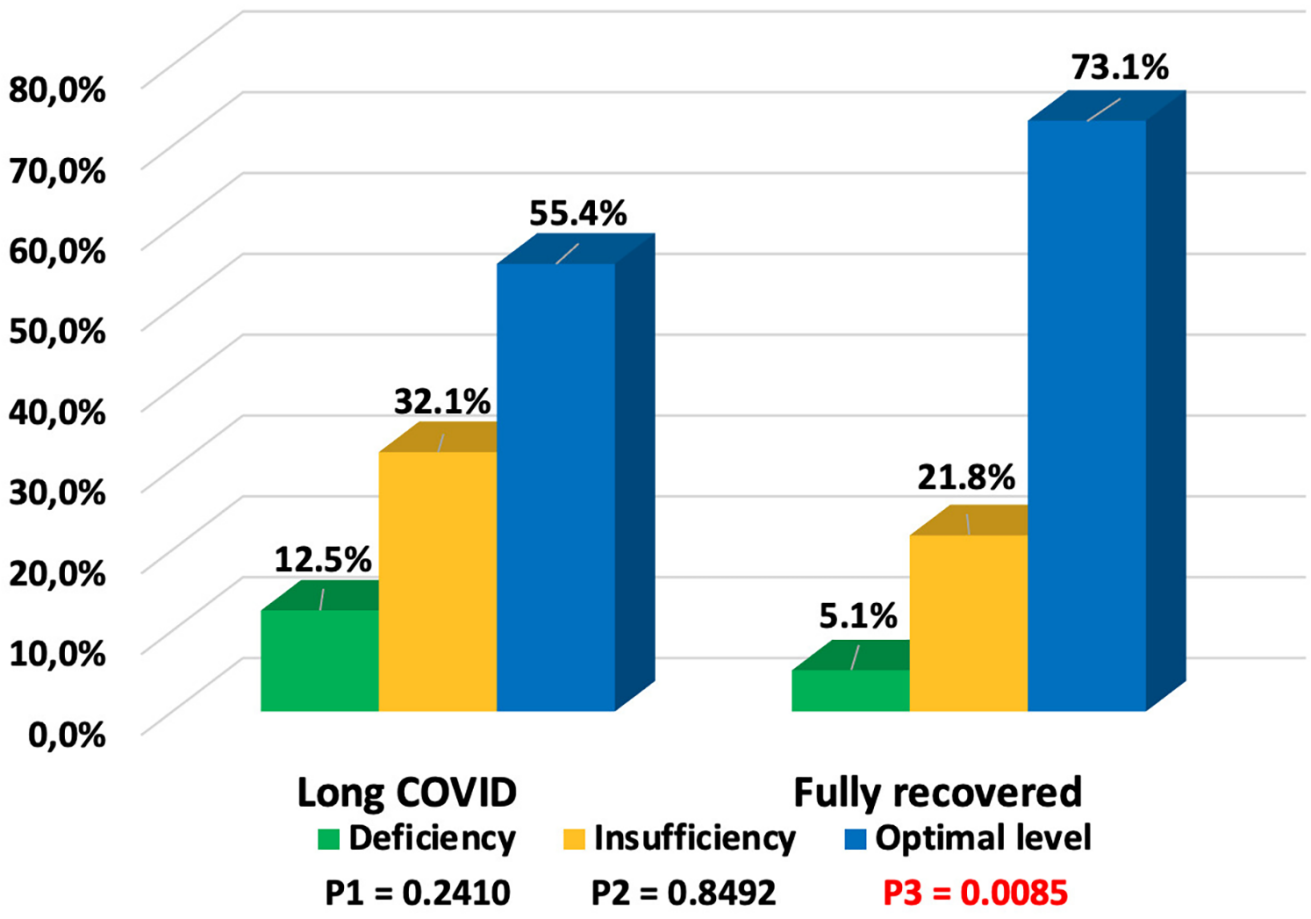
Vitamin D status in children with long COVID and fully recovered (P1—value between groups of children with long COVID and fully recovered with vitamin D deficiency, P2—value between groups of children with long COVID and fully recovered with vitamin D insufficiency, P3—value between groups of children with long COVID and fully recovered with optimal vitamin D levels).

The vitamin D status in patients with long COVID and the symptoms of long COVID based on vitamin D status are shown in Table 2. Vitamin D deficiency was observed in 7 (12.5%) children, insufficiency in 18 (32.1%), and optimal levels in 31 (55.4%) children who subsequently developed long COVID symptoms, while among recovered children, optimal vitamin D levels were found in 57 (73.1%). Long COVID was more frequently observed in children with vitamin D deficiency/insufficiency) (Figure 5), and this difference was statistically significant (*p* = 0.0331). The odds of developing long COVID were 2.2 times higher (OR = 2.1889, 95% CI: 1.0585–4.5267; *p* = 0.0346) in children with vitamin D deficiency/insufficiency compared to those with optimal vitamin D levels. It should be noted that vitamin D status in children with symptoms of both long COVID and those who recovered depended on the age of the children. Deficiency/insufficiency of vitamin D was observed in 15/18 (83.3%) children over 6 years old with long COVID symptoms and in 11/14 (78.5%) children who recovered (*p* = 0.7321). The median concentration of 25(OH)D in patients with long COVID symptoms was lower (34.52 ng/ml; IQR: 23.69; 67.31 ng/ml) compared to those who recovered (44.52 ng/ml; IQR: 28.65; 70.03 ng/ml), but the difference was not statistically significant, *p* = 0.1451.

**Table 2:** Symptoms of long COVID in hospitalized children depends on vitamin D status.

Characteristics	Vitamin D deficiency/insufficiency	Optimal vitamin D level	*P*
	*n* (%)	*n* (%)	
Long COVID	25/46 (54.3)	31/88 (35.2)	**0** **.** **0331**
General	16/25 (64.0)	21/31 (67.7)	0.7687
Neurological	20/25 (80.0)	13/31 (41.9)	**0**.**0040**
Gastroenterological	1/25 (4.0)	8/31 (25.8)	**0**.**0272**
Cardiological	1/25 (4.0)	1/31 (3.2)	0.8767
Musculoskeletal	4/25 (16.0)	0/31	**0**.**0208**
Frequent acute respiratory infections	6/25 (24.0)	8/31 (25.8)	0.8767

Statistically significant values are highlighted in bold.

General and cardiological symptoms were observed equally frequently in both groups, regardless of vitamin D status. Neurological manifestations were almost twice as common in children with vitamin D deficiency/insufficiency compared to those with optimal levels (*p* = 0.0040). Low vitamin D levels also affected musculoskeletal manifestations (16% vs. 0%, *p* = 0.0208). The odds of developing neurological symptoms of long COVID were 5.5 times higher (OR = 5.5385, 95% CI: 1.6480–18.6133; *p* = 0.0056) in children with vitamin D deficiency/insufficiency compared to those with optimal vitamin D levels. For musculoskeletal symptoms, OR = 13.1860, 95% CI: 0.6746–257.7413; *p* = 0.0890. In contrast, gastroenterological symptoms were more frequently observed in patients with normal vitamin D levels (*p* = 0.0272). The increased incidence of respiratory diseases was nearly the same in both groups of children.

## Discussion

Our study revealed a decrease in vitamin D levels in one-third of children with COVID-19. However, findings from other studies regarding the prevalence of hypovitaminosis D among children with acute SARS-CoV-2 infection remain contradictory. Bayramoğlu et al. (29) reported hypovitaminosis D in 35.4% of hospitalized children with mild and moderate disease. This aligns with our findings, as most children in our cohort had mild COVID-19. In contrast, Bayrak et al. (34) found a higher prevalence of vitamin D deficiency or insufficiency (67.1%) among hospitalized children with COVID-19. Furthermore, the average 25(OH)D concentration in their study was significantly lower compared to that of our cohort.

Vitamin D status is influenced not only by the disease itself but also by various external factors. In our cohort, vitamin D status was notably age-dependent, with vitamin D deficiency or insufficiency observed in 73% of children over six years of age. Other studies have also highlighted an age-related dependence of vitamin D levels in hospitalized COVID-19 patients (35), with adolescents being more susceptible to developing hypovitaminosis D. This increased susceptibility among adolescents may stem from their tendency toward sedentary lifestyles, particularly during extended periods of staying at home, with greater access to computers, televisions, and smartphones (36, 37).

The COVID-19 pandemic has contributed to prolonged home confinement for both children and adults, significantly reducing sun exposure time (38, 39). Epidemiological studies have indicated that the pandemic has altered vitamin D levels, particularly among preschool- and school-aged children (40).

Our study revealed differences in mean 25(OH)D concentrations based on COVID-19 severity and the length of hospital stay among children with different vitamin D status. These findings align with other studies that report a direct impact of vitamin D levels on symptoms, severity, and outcomes of COVID-19 (28–30, 41).

Notably, among our patients, pneumonia was diagnosed in only 8 children (4.9%), and 6 (75.0%) of whom had hypovitaminosis D. Nicolae et al. (42) confirmed that hypovitaminosis D could contribute to the development of pneumonia due to its significant influence on immunological processes. Regression analysis in another study also identified low vitamin D levels as a risk factor for developing respiratory distress (41). Furthermore, Kosmeri et al. (43) demonstrated an association between vitamin D insufficiency and the severity of COVID-19 in adults. Their study highlighted 25(OH)D concentration as an independent risk factor for COVID-19 infection and the likelihood of requiring hospitalization.

Other studies have demonstrated the impact of vitamin D status on COVID-19 mortality, especially in adult patients (30, 44, 45). A population-based study revealed a negative correlation between 25(OH) vitamin D average levels and COVID-19 mortality in 19 European countries (44). Seal et al. demonstrated that 25(OH)D concentrations were associated with COVID-19-related hospitalization and mortality in a cohort of veterans (45). A meta-analysis also declared an association between COVID-19 severity and mortality and low serum vitamin D levels (46).

The association between vitamin D deficiency and disease severity or increased mortality is likely related to impaired immune responses. Vitamin D plays a critical role in supporting the immune system. It modulates innate and adaptive immune responses, enhancing the body's ability to fight infections and reducing excessive inflammatory responses (24, 27, 36). Through its effects on immune cells such as macrophages, dendritic cells, and T cells, vitamin D promotes the production of antimicrobial peptides like cathelicidins and defensins, which are vital for neutralizing pathogens (25). Furthermore, vitamin D stabilizes endothelial function and regulates vascular permeability, reducing the risk of cytokine storm and associated complications often observed in severe infections like COVID-19 (29).

An analysis of laboratory characteristics revealed that the median levels of neutrophils and the neutrophil-to-lymphocyte ratio were significantly higher in children with low vitamin D levels (*p* = 0.0021; *p* < 0.0001, respectively), whereas median levels of lymphocytes and platelets were significantly lower in this group of patients (*p* = 0.0044; *p* = 0.0071, respectively). Alpcan et al. (41) reported a positive correlation between vitamin D levels and leukocyte, lymphocyte, and platelet counts. Similarly, another study found that lower vitamin D levels were associated with increased clinical severity and more pronounced inflammatory markers (29). In our study, CRP was elevated in 55.6% of children with COVID-19 and vitamin D deficiency/insufficiency; however, no statistically significant difference was observed compared to patients with optimal vitamin D levels. Ferritin levels were elevated in only two patients, and no dependence on vitamin D status was identified. By contrast, another study demonstrated significantly higher levels of CRP and ferritin in adult patients with low vitamin D levels (47). Moreover, elevations in CRP, fibrinogen, and lymphopenia were more frequently observed in cases of vitamin D deficiency rather than insufficiency (29). In our cohort, the proportion of children with vitamin D deficiency was relatively small, comprising only 8.0% of the population, while the majority exhibited vitamin D insufficiency. This distribution may explain the lack of statistically significant differences in certain inflammatory markers.

We also observed changes in coagulation markers, specifically increased PT and fibrinogen levels (*p* = 0.0058; *p* = 0.0037, respectively). Our previous research demonstrated age-related characteristics of coagulation markers in children with COVID-19 (48). Other studies have also reported a relationship between vitamin D levels and thrombotic complications in COVID-19 patients (47, 49–51). Cooper et al. (50) noted that vitamin D activation reduces the risk of respiratory infections and decreases coagulation and thrombosis.

Increased IgA levels were nearly twice as common, IgM levels were 1.5 times more frequent, IgE levels were 3.7 times more frequent, and changes in IgG levels were 1.8 times more frequent in children with vitamin D deficiency/insufficiency compared to those with optimal levels. Our findings align with results from other researchers, who demonstrated that the immunomodulatory effects of vitamin D are associated with the inhibition of B-cell proliferation, blockage of their differentiation, and a significant decrease in immunoglobulin secretion (52).

On one hand, the more frequent elevation of immunoglobulin levels in cases of vitamin D deficiency/insufficiency may be linked to a more severe course of illness, as was also shown in our study. Specifically, Peraire et al. (53) investigated the relationship between circulating immunoglobulins (IgA, IgG, IgM) and COVID-19 pneumonia. They established that IgM, IgA, and IgG concentrations were significantly higher in patients with COVID-19 pneumonia (mild, severe, and critical forms) compared to those in the ambulatory group (*P* ≤ 0.001).

On the other hand, increased immunoglobulin production in children with reduced vitamin D levels may potentially contribute to the development of autoimmunity and/or symptoms of long COVID (8, 13, 26). Notably, several studies have demonstrated a rise in autoimmune diseases following the COVID-19 pandemic (54).

In our study, symptoms of long COVID were observed in 41.8% of hospitalized children. The prevalence of patients with long COVID symptoms is highly variable, ranging from 3.4% in symptomatic patients (55) to 81.4% in hospitalized patients (56). Numerous factors can influence the prevalence of long COVID, including age, study cohort, follow-up duration, comorbidities, COVID-19 vaccination status, etc. Rao et al. reported that the frequency of at least one systemic, syndromic, or drug-induced sign of post-acute complications of SARS-CoV-2 infection was 41.9% among children with a positive test for the virus (57), which aligns with the results of our study. The authors highlighted a higher prevalence of long COVID symptoms among children under 5 years of age and those with comorbidities. The high prevalence of comorbid conditions in our cohort (46.3%) and the predominance of children under 6 years of age (77.2%) could explain the high frequency of long COVID symptoms. It should also be noted that we studied the presence of symptoms specifically in hospitalized patients, which may have contributed to the high frequency of long COVID in our findings.

Our study showed that children with vitamin D deficiency or insufficiency had more than twice the risk of developing long COVID compared to those with optimal vitamin D levels (OR = 2.1889, *p* = 0.0346). These findings align with results from other studies. For instance, lower 25(OH) vitamin D concentrations were reported six months after the acute phase of the disease in adults with long COVID compared to those who fully recovered from coronavirus infection (20.1 vs. 23.2 ng/ml, *p* = 0.0300) (58). Similarly, Chen et al. demonstrated that vitamin D deficiency was associated with delayed recovery in adult patients with long COVID (59). A study by Guerrero-Romero et al. highlighted a threefold increase in the risk of developing long COVID in adult patients with insufficient vitamin D and magnesium levels (60). However, several investigations involving adult populations did not find a significant association between low serum vitamin D levels and long COVID (59, 61, 62). Pizzini et al. conducted a prospective, multicenter study on the long-term sequelae of COVID-19 and their association with 25(OH)D concentrations (63). While vitamin D deficiency was commonly observed among COVID-19 patients, it was not linked to long-term disease outcomes. Similarly, other researchers found reduced concentrations of vitamins D, A, and E in adult patients with COVID-19 but reported no significant impact on the development of long COVID symptoms (64).

In children, research has primarily focused on vitamin D status concerning multisystem inflammatory syndrome (MIS-C) associated with COVID-19. Studies have shown that children with MIS-C had significantly lower vitamin D levels than those in the non-MIS-C group (65), and vitamin D deficiency was found in 72% of children with MIS-C (31).

The impact of vitamin D levels on long COVID symptoms in children has also been analyzed. We observed an almost twofold increase in the frequency of neurological symptoms in patients with vitamin D deficiency (*p* = 0.0040) and a higher incidence of musculoskeletal symptoms (*p* = 0.0208). However, the odds ratio indicated only an increased risk of neurological symptoms. Another study found lower 25(OH)D levels in patients with neurocognitive symptoms six months after recovering from COVID-19 (60). A multivariable regression analysis conducted by Townsend et al. showed no relationship between persistent fatigue and reduced exercise tolerance following COVID-19 (66).

Given vitamin D's ability to modulate both immune and nerve cells, its role in neuroimmune modulation, anti-inflammatory action, neuroprotection, and endothelial function suggests that it could play a positive role in preventing and managing neuropsychiatric, neuroinflammatory, and other processes in long COVID (67, 68). However, further research is needed to determine optimal dosages and the duration of treatment.

### Strengths and limitations of the study

This study provides valuable insight into the role of vitamin D in pediatric COVID-19 patients, an area where data remains limited. Most studies have focused on adults, making this work a significant contribution to understanding long COVID in children. The study includes a prospective cohort design with thorough follow-up, allowing for detailed observations of long COVID symptoms in relation to vitamin D status.

However, the study has several limitations. The sample size, particularly for children with long COVID, is relatively small, which may affect the generalizability of the results. The study population was drawn from a tertiary-level pediatric hospital, meaning the patients may differ from those in other pediatric hospitals. Additionally, no control group of healthy children did not contract COVID-19, which could have provided additional comparative data on vitamin D status in non-infected populations. The study was conducted during a specific time frame, and seasonal variations in sunlight exposure, which could affect vitamin D levels, were not fully accounted for. Another limitation is the method used to measure vitamin D levels—ELISA—which is less sensitive and specific compared to chemiluminescence and mass spectrometry, the internationally recommended methods.

## Conclusion

The 25(OH)D concentrations in children with COVID-19 depended on their age. Vitamin D deficiency/insufficiency was observed in 73% of children over 6 years of age and 21.6% of children under 6 years of age with COVID-19. The presence of comorbid conditions, particularly undernutrition and obesity, affected the vitamin D status in children with COVID-19. Vitamin D influenced the COVID-19 severity and duration of hospitalization. There was an increased risk of developing long COVID in children with vitamin D deficiency/insufficiency, and its impact on the development of neurological symptoms associated with long COVID was established. Further research is needed to determine independent predictors of long COVID development.

## Data Availability

The raw data supporting the conclusions of this article will be made available by the authors, without undue reservation.
